# Retraction Note: Cross-HLA targeting of intracellular oncoproteins with peptide-centric CARs

**DOI:** 10.1038/s41586-023-06731-z

**Published:** 2023-11-08

**Authors:** Mark Yarmarkovich, Quinlen F. Marshall, John M. Warrington, Rasika Premaratne, Alvin Farrel, David Groff, Wei Li, Moreno di Marco, Erin Runbeck, Hau Truong, Jugmohit S. Toor, Sarvind Tripathi, Son Nguyen, Helena Shen, Tiffany Noel, Nicole L. Church, Amber Weiner, Nathan Kendsersky, Dan Martinez, Rebecca Weisberg, Molly Christie, Laurence Eisenlohr, Kristopher R. Bosse, Dimiter S. Dimitrov, Stefan Stevanovic, Nikolaos G. Sgourakis, Ben R. Kiefel, John M. Maris

**Affiliations:** 1 Division of Oncology and Center for Childhood Cancer Research, Children’s Hospital of Philadelphia, Philadelphia, PA USA; 2 Myrio Tx, Melbourne, Victoria Australia; 3 Department of Biomedical and Health Informatics, Children’s Hospital of Philadelphia, Philadelphia, PA USA; 4 University of Pittsburgh, Pittsburgh, PA USA; 5 University of Tubingen, Tubingen, Germany; 6 Department of Chemistry and Biochemistry, University of California Santa Cruz, Santa Cruz, CA USA; 7 Massachusetts Institute of Technology, Cambridge, MA USA; 8 Department of Pathology and Lab Medicine, Children’s Hospital of Philadelphia, Philadelphia, PA USA; 9 Perelman School of Medicine at the University of Pennsylvania, Philadelphia, PA USA

**Keywords:** Cancer immunotherapy, MHC class I

Retraction to: *Nature* 10.1038/s41586-021-04061-6 Published online 3 November 2021

The authors are retracting this manuscript because of an important experimental error we detected. The manuscript reported tetramer staining experiments showing that 10LH can interact with multiple HLA allotypes including the HLA-B*14:02/PHOX2B peptide complex (Fig. 1a, 3c). Following the publication of our manuscript, a new high-resolution structure of the HLA-A*24:02/PHOX2B peptide/10LH scFv complex by X-ray crystallography alerted us to the fact that HLA-B*14:02 data were difficult to explain. We therefore used more detailed biophysical characterization by Surface Plasmon Resonance and determined that HLA-B*14:02/PHOX2B does not interact with 10LH up to the millimolar range, consistent with the protein interfaces determined in the X-ray structure. Moreover, HLA-B*14:02 is sub-optimal for binding to the PHOX2B peptide, as shown by protein refolding experiments. Subsequent analysis of the protein samples used to perform the original tetramer staining experiments by mass-spectrometry revealed contamination with HLA-A*23:01 protein. The contamination was present in the protein refolding step, and therefore the results presented in Extended Data Fig. 14c also correspond primarily to the HLA-A*23:01/PHOX2B complex. This affects the title, abstract, and a number of statements in the article, as well as Figure 3. The authors apologize for any inconvenience or confusion to readers caused by the mistake, which does not impact the ability of 10LH to recognize PHOX2B presented on two common HLA allotypes in a highly peptide-specific manner, as reported in the original version of our manuscript, nor any other data in the manuscript. In addition, we also note a typographical error in Fig. 2D: The MYO7B peptide sequence should be SGFPIRYTF instead of RQSPWRIYF, as published. All the authors agree with the retraction. A revised manuscript has been subsequently submitted and published after peer review^1^.
